# In Vitro Comparison of Optical Properties Between Single-Shade and Conventional Composite Resin Restorations

**DOI:** 10.7759/cureus.57664

**Published:** 2024-04-05

**Authors:** Waad F Khayat

**Affiliations:** 1 Department of Restorative Dentistry, Umm Al-Qura University, Makkah, SAU

**Keywords:** color change, spectrophotometer, single-shade restoration, refractive index, omnichroma, composite resin, color stability, color matching, color difference, dental esthetics

## Abstract

Introduction: The recently developed single-shade composite resin (using the concept of structural coloring) is a promising option to eliminate the process and subjectivity of shade selection. However, current evidence of its performance is still insufficient. This study aimed to evaluate the color matching, color stability, and refractive index (RI) of single-shade restorations compared with conventional composite resin.

Methods: This in vitro study was conducted on 48 extracted maxillary premolars. The teeth were sorted into three tooth shade groups (B2, A3, and A3.5). Each group included two subgroups based on the materials used: Omnichroma (OM; Tokuyama Dental, Tokyo, Japan) and Filtek Z350 (FT; 3M ESPE, St. Paul, MN, USA). Buccal and lingual Class V round cavities were prepared and restored according to the randomly assigned subgroups, with the test single-shade material (OM) on one surface and the matching conventional control material (FT) of the proper shade on the other surface of each tooth. Color matching was evaluated by visual assessment and a spectrophotometer. All specimens were distributed into four staining groups and immersed in various staining beverages (black coffee, Arabic coffee, Coca-Cola, and distilled water) for four weeks. Color changes were assessed using a spectrophotometer. Four groups of disc-shaped specimens (single-shade, B2, A3, and A3.5) were fabricated to evaluate the RI using a refractometer. Statistical analyses were performed to compare the materials.

Results: The mean value of the color difference (ΔE) of single-shade restorations was significantly higher than that of paired conventional restorations in all shade groups. The highest mean ΔE was in subgroup A3.5-OM (8.84 ± 2.39) and showed significantly less color matching than other OM subgroups (p=0.009). The visual assessment showed comparable and acceptable color matching in all subgroups except A3.5-OM (p=0.006). There was a significant color change after the staining test within and across staining groups, but the color change was comparable between the OM and FT subgroups. The RI of single-shade material was significantly higher than conventional material (p<0.05).

Conclusion: The color matching of single-shade restorations was affected by tooth shade. Its color stability was influenced by various staining substances but was similar to conventional restorations.

## Introduction

Dental shade selection is a challenging process in clinical practice. The esthetic failure of dental restorations due to a color mismatch may require replacement of the restoration, which brings additional costs and working time and may cause patient frustration. Over the years, great efforts have been made to understand the complex interactions of light with tooth structure and restorations to reduce challenges related to the subjectivity of shade selection and human color perception and to overcome the limitations of currently available shades of resin and shade guides [1].

The demand for a simplified shade selection and matching process led to the introduction of a single-shade composite resin material in 2019, using the concept of structural coloration produced by the interaction of light with the microstructure of the material instead of chemical coloration produced by pigments [2,3]. The major advantage of this recently introduced material is the ability to use one shade of composite resin for a wide range of direct restorations of various tooth shades. This minimizes chairside time, reduces waste of materials, saves money (supplies of different shades are no longer needed), and reduces reliance on a subjective shade selection process [4].

Many criteria should be considered in esthetic restorative materials, such as accurate color matching, high polishability, matching refractive index (RI), and enamel-like translucency. Moreover, color stability and resistance to staining are crucial in determining the long-term durability of restorations [5]. Current evidence regarding the properties of the recently developed single-shade resin material in the dental literature is still insufficient, and the influence of various forms of aging on the performance of this material compared to the commonly used conventional nano-composite resin (developed in multiple enamel, body, and dentin shades) is not clear. Therefore, extensive investigations of the clinical-related esthetic properties and performance of this new material are necessary to provide data for evidence-based practice.

The primary aim of the current study was to compare color matching between single-shade composite resin restorations and their paired conventional restorations of different shades (B2, A3, and A3.5) using visual scoring (VS) and spectrophotometer assessment of color difference (ΔE). In addition, this study aimed to evaluate the influence of staining beverages (colored carbonated drinks and coffee) on the color stability of the tested dental materials. Also, the study aimed to measure the RI of both restorative materials. The first null hypothesis was as follows: the color-matching ability of single-shade composite resin is similar to conventional composite resin and is not influenced by tooth shade. The second hypothesis was as follows: the color stability of single-shade restoration is similar to that of conventional material and is not affected by chemical aging using various staining beverages. The third null hypothesis was as follows: single-shade and conventional composite resin materials have similar RI.

## Materials and methods

Materials and study design

**Figure 1:**
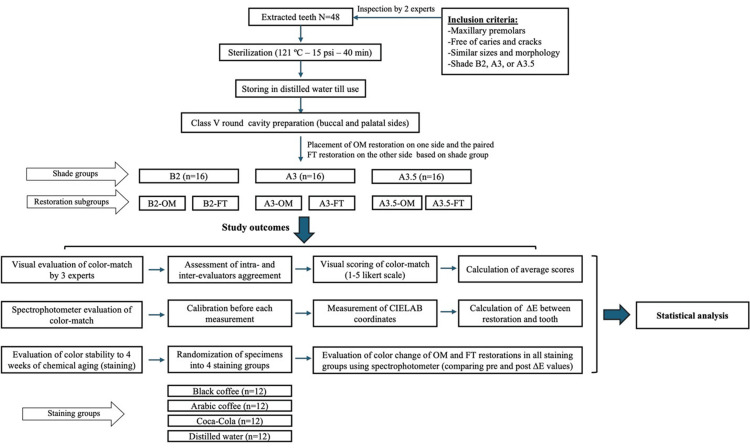
Study design and methods of the present in vitro study

Ethical approval for this in vitro study was obtained from the Institutional Review Board of Umm Al-Qura University (approval number: HAPO-02-K-012-2021-09-752). The types, composition, and manufacturer data of the materials used are shown in Tables 1-2. After obtaining the patients’ consent, a total of 48 freshly extracted human maxillary premolars were collected, cleaned, sterilized, and stored in distilled water until use [6]. The included teeth were intact, had comparable dimensions, and had B2, A3, or A3.5 tooth shades. These three tooth shades were selected for color-match evaluation based on the order of color value in the Vita Classical Shade Guide to represent common shades and various levels of value and chroma. The assessment of inclusion criteria and tooth shade was done visually by two expert evaluators under standardized conditions. The teeth were labeled and sorted based on tooth shade into three shade groups: Group B2 (n=16), Group A3 (n=16), and Group A3.5 (n=16). Each group included two subgroups (OM and FT) based on restorative materials: Omnichroma (OM; Tokuyama Dental, Tokyo, Japan) and Filtek Z350 (FT; 3M ESPE, St. Paul, MN, USA), respectively, as shown in Figure 1.

**Table 1:** Dental composite resin materials used in the study Information disclosed by manufacturers on material safety data sheet

Material	Omnichroma	Filtek Z350
Manufacturer	Tokuyama Dental, Tokyo, Japan	3M ESPE, Saint Paul, MN, USA
Resin composition	Urethane dimethacrylate (UDMA), triethylene glycol dimethacrylate (TEGDMA), mequinol, dibutyl hydroxytoluene, and ultraviolet ray absorbers	Bisphenol A diglycidyl dimethacrylate (bis-GMA), urethane dimethacrylate (UDMA), triethylene glycol dimethacrylate (TEGDMA), and Bis-EMA ethoxylated bisphenol A dimethacrylate (PEGDMA)
Fillers composition	Uniform-sized supra‐nano spherical filler (260 nm spherical SiO_2_‐ ZrO_2_)	Non-agglomerated/non-aggregated silica filler (20 nm), non-agglomerated/non-aggregated zirconia filler (4–11 nm), and aggregated zirconia/silica cluster filler (comprised of 20 nm silica and 4–11 nm zirconia particles), cluster particle size 0.6-10 microns
Filler load	79% by weight 68% by volume	78.5% by weight 63.3% by volume
Shade	Universal	Body shades A2B, A3.5B, B2B

**Table 2:** Compositions of substances used for staining test Information disclosed by manufacturers

Staining beverage	Composition	Manufacturer
Nescafe classic (15 g of coffee: 250 ml of boiling water)	Sugar, glucose syrup, instant coffee (11%), palm kernel oil, soluble fiber, skimmed milk powder (0.7%), MILK protein, salt, stabilizers, lactose (milk), acidity regulator, emulsifiers, natural flavorings, milk fat, color	Nestle, Saudi Arabia
Arabic coffee (15 g of coffee: 250 ml of boiling water)	Instant Saudi coffee, cardamom, cloves, nondairy coffee creamer, saffron, used as hot coffee	BajaFood Industrial Co., Saudi Arabia
Coca-Cola	Sugar, caramel, caffeine, orthophosphoric acid, water	Coca-Cola, USA
Distilled water	Distilled water	Pharmapack, Egypt

Preparation of specimens

Round-shaped cavities (3 mm diameter x 1.5 mm depth) were prepared on the buccal and palatal surfaces of all teeth located 3 mm coronal to the cementoenamel junction using a jig to fix the specimens, a water-resistant pen to draw outlines on the predetermined position, a high-speed round carbide bur for every eight preparations, and a perio probe to check dimensions. Each tooth received two restorations; the test cavity was restored on the buccal or palatal surface with OM material in a random base, while the matching control cavity on the other side received FT composite resin of the appropriate corresponding tooth shade. A celluloid matrix was placed on top of the restoration before curing to eliminate the need for polishing. The teeth were stored in distilled water for 24 hours at room temperature before testing. All preparations and restorations were done by a single operator following the manufacturers’ instructions under standardized conditions.

Four groups (n=12) of disc-shaped specimens (10 mm in diameter and 1.5 mm in thickness) were fabricated using a specific ring-shaped stainless steel mold. The first group was made of OM, while the second, third, and fourth groups were made of FT (body shades B2B, A3B, and A3.5B, respectively). All specimens were immersed in distilled water for 24 hours in a dark environment at room temperature before evaluating the RI.

Evaluation of color matching

Instrumental evaluation of color matching was recorded using a spectrophotometer (Vita Easy Shade Advance 4, Zahnfabrik, Bad Sackingen, Germany) to measure the CIELAB coordinates (L, a, and b) of the restorations and the surrounding tooth structure at a wavelength of 555 nm. Calibration was performed before each measurement, and each tooth specimen was measured by holding the probe tip at 90° to the selected areas (center of the restoration, tooth structure 1 mm to the right, left, occlusal, and gingival to the restoration) under standardized conditions. The average value of the measured areas on the tooth structure of each specimen was calculated. The values of ∆L, ∆a, and ∆b between the restoration and the surrounding tooth structure were determined. ∆E was calculated according to the following equation: ΔE= ((ΔL)^2^ + (Δa)^2^ + (Δb)^2^)^1/2^, where ΔL= the differences between L values of restoration and tooth, Δa= the differences between values of restoration and tooth, and Δb= the differences between b values of restoration and tooth.

A visual evaluation of the color matching of restorations was performed by three expert dentists with normal color vision. The evaluators were trained to use VS values expressed numerically (1=totally unacceptable, 2=hardly acceptable, 3=acceptable, 4=small difference, and 5=no color difference). Inter- and intra-agreements between evaluators were checked. The shade matching was evaluated using a D65 light bulb with a color temperature of 6500 kelvin to provide daylight-like illumination and a color rendering index >90 to enhance the accuracy and the trueness of the color displayed (Colour Viewing Lamp F15T8/CM65, Graphic Technology Ltd., Mirfield, UK). A holder was used to provide a standardized 45° angle and a fixed distance of 25 cm between the evaluators’ eyes and the specimens with a light gray background. The evaluators were blind to the type of restoration being evaluated, and they assessed the specimens in random order. The average of the evaluators’ scores (VS) was calculated for each restoration.

Evaluation of color stability

The specimens were divided equally into four staining groups (n=12) according to the staining media (black coffee, Arabic coffee, Coca-Cola, and distilled water). The specimens were then stored in these solutions in an incubator at 37°C for four weeks (equivalent to approximately two years and six months of clinical aging) [7]. The staining solutions were stirred daily and replaced twice a week to avoid microbial contagion. At the end of the staining test, the specimens were removed from the solutions, washed thoroughly, and gently dried with air. Spectrophotometer measurements were taken for each specimen before and after the staining test to determine the color change.

Evaluation of refractive index

Refractive indices were measured for all disc-shaped specimens using a refractometer (Refractive Index Meter II, Presidium Instruments Ltd., Singapore). Two readings were taken for each specimen, and the average was calculated.

Sample size and statistical analysis

The sample size was calculated (G*power version 3.1.9, Heinrich-Heine-Universität Düsseldorf, Germany). A sample size of n=12 per group was adequate (Type I error rate = 5%, power = 80%, and effect size = 0.5). A Shapiro-Wilk test was done to check the normality of the distribution. Descriptive statistics were presented, and statistical analysis tests were applied (one-way ANOVA, independent samples t-test, and dependent samples t-test). Non-parametric tests (Wilcoxon Signed Rank and Kruskal-Wallis) were used when indicated. A post hoc test with Bonferroni correction was done for multiple comparisons. A p-value ≤0.05 was considered statistically significant. SPSS Statistics version 26.0 (IBM Corp. Released 2019. IBM SPSS Statistics for Windows, Version 26.0. Armonk, NY: IBM Corp.) was used for the statistical analyses.

## Results

Color matching

The mean ΔE of OM restorations showed statistically significantly higher values than the paired FT restorations of the corresponding color in all shade groups (B2, A3, and A3.5), as shown in Table 3. A Kruskal-Wallis test performed to compare the mean ΔE between OM subgroups (B2-OM, A3-OM, and A3.5-OM) showed a statistically significant difference between them (p=0.01). Pairwise comparison with Bonferroni correction showed that the subgroup A3.5-OM (which recorded the highest mean ΔE among all subgroups) had significantly less color matching than the B2-OM and A3-OM subgroups (p=0.009).

**Table 3:** Comparison of color difference (ΔE) between single-shade restorations and their paired conventional composite resin restorations * A dependent sample t-test was performed to compare the difference means of ΔE between paired materials within each group at p<0.05

Group	Subgroup	Mean ± SD	Difference mean ± SD	t	p-value^*^
B2	B2-OM	6.04 ± 0.96	0.94 ± 1.44	2.10	0.028
	B2-FT	5.10 ± 1.22			
A3	A3-OM	7.44 ± 2.15	2.27 ± 1.92	3.92	0.003
	A3-FT	5.16 ± 1.36			
A3.5	A3.5-OM	8.84 ± 2.39	3.12 ± 1.98	5.21	0.001
	A3.5-FT	5.72 ± 2.78			

A Kappa test was used to determine intra-evaluator and inter-evaluator variability, which were 0.91 and 0.79, respectively. The visual scores of all OM and FT restorations ranged between 4 and 5 (detecting small to no perceptible differences in color match). The mean VS values of OM restorations were comparable to the mean values of FT restorations in groups B2 and A3 (no statistically significant difference was detected between the types of restorations), but the difference in color matching was statistically significant between OM and FT restorations in group A3.5 (Table 4).

**Table 4:** Comparison of the visual score (VS) of single-shade restorations and their paired conventional composite resin restorations * A Wilcoxon Signed Rank test was performed to compare visual scores between paired materials within each shade group at p<0.05

Group	Subgroup	Mean ± SD	Median	Interquartile range	p-value^*^
B2	B2-OM	4.61 ± 0.33	4.6	0.00	0.739
	B2-FT	4.67 ± 0.37	4.6	0.67	
A3	A3-OM	4.52 ± 0.27	4.6	0.33	0.564
	A3-FT	4.58 ± 0.26	4.6	0.33	
A3.5	A3.5-OM	4.24 ± 0.39	4.0	0.67	0.006
	A3.5-FT	4.67 ± 0.26	4.6	0.67	

Color stability

The color changes of the restorations after immersion in different beverages were measured. There was a statistically significant color change in both OM and FT materials after immersion in staining solution across and within staining groups (Tables 5-6). The highest color change was caused by black coffee in both types of restorations. However, the color change was comparable between the OM and FT materials (no significant difference between them) in all staining groups (Table 7).

**Table 5:** Comparison of color difference (ΔE) across staining groups * A one-way ANOVA was performed to compare the ΔE means and standard deviation across staining groups for each material at p<0.05. A post hoc test with Bonferroni correction was performed for multiple comparison tests. Different superscript letters indicate a statistically significant difference between the staining groups at p<0.05

Restorative material	Staining	Black coffee	Arabic coffee	Coca-Cola	Distilled water	F test	p-value^*^
Omnichroma	Before staining	7.61 ± 2.31	6.80 ± 2.25	7.80 ± 2.06	6.66 ± 2.08	5.83	0.040
	After staining	29.02 ± 3.89^a^	13.52 ± 5.52^b^	10.54 ± 2.39^c^	6.81 ± 2.33^d^	74.08	0.001
Filtek Z350	Before staining	5.70 ± 2.25	5.09 ± 1.78	5.28 ± 1.67	5.11 ± 2.09	1.72	0.180
	After staining	29.14 ± 4.27^e^	16.32 ± 2.57^f^	11.51 ± 3.96^g^	5.87 ± 1.99^h^	69.59	0.001

**Table 6:** Comparison of pre- and post-color changes within staining groups * A dependent samples t-test was performed to compare the ΔE means before and after staining within staining groups for each material at p<0.05

Restorative material	Staining groups	Mean difference of color change (ΔE)	t	p-value^*^
Omnichroma	Black coffee	21.41	15.86	0.001
	Arabic coffee	6.72	3.91	0.004
	Coca-Cola	5.26	5.06	0.001
	Distilled water	0.15	2.52	0.030
Filtek Z350	Black coffee	23.44	13.96	0.001
	Arabic coffee	11.23	11.81	0.001
	Coca-Cola	6.23	2.93	0.019
	Distilled water	0.76	2.19	0.004

**Table 7:** Comparison of color changes between single-shade and conventional material among staining groups * An independent samples t-test was performed to compare ΔE means after staining between OM and FT restorative materials at p<0.05

Staining group	Mean difference of color change (ΔE)	t	p-value^*^
Black coffee	0.12	0.07	0.948
Arabic coffee	2.8	0.56	0.163
Coca-Cola	0.97	0.45	0.659
Distilled water	-0.94	0.44	0.893

Refractive index

There was a statistically significant difference in RI between the groups (p=0.017). A pairwise test for multiple comparisons revealed that OM material had a significantly higher mean RI (1.579 ± 0.01) than FT groups, showing values of 1.527 ± 0.02, 1.533 ± 0.006, and 1.537 ± 0.01 for shades B2, A3, and A3.5, respectively (p<0.05).

## Discussion

This study compared the color matching and color stability between two types of restorative materials (OM and FT) placed in extracted teeth of different shades. The first null hypothesis was rejected, as the findings confirmed that single-shade material showed a statistically significant lower color-matching ability to surrounding tooth structure (higher ΔE) than the corresponding multi-shade restorations in all shade groups. Prior dental literature has shown controversial results; some studies support this finding of a perceptible color mismatch with single-shade restoration (reporting similar or higher mean ΔE compared with our results) [4,8-12], while other studies showed better color matching with single-shade material than with conventional composite resin [13-15]. Alhamdan et al., who assessed the same materials on various tooth shades, reported similarly significantly lower color-matching ability in single-shade restoration than with conventional composite resin, but their measurements of the mean ΔE of the materials were remarkably lower than ours [16]. The variability of numeric results between studies could be attributed to non-standardized experimental parameters, including applying different cavity designs (Class I or III vs. V), depth of cavity (1.5-3 mm), types of specimens (acrylic vs. natural teeth), and different types and settings of instrumental evaluation methods [17].

The study also found that the color-matching ability of single-shade restorations is significantly influenced by the shade of the tooth. OM restorations revealed significantly better color matching in teeth with a lighter shade (B2) than in darker teeth (A3.5). Similar results from previous studies showed that color-matching ability is shade-dependent and influenced by tooth color (the higher the color value of the tooth, the less ΔE between the restoration and tooth) [2,8,10,18,19]. Our finding of a significantly detectable color mismatch by visual evaluation between the OM and FT restorations in group A3.5 corresponded with the measurements obtained by spectrophotometer, showing that the highest mean ΔE for OM restorations was in group A3.5 (ΔE=8.8 ± 2.4) compared with other shade groups. This result is supported by a previous study that also found that the visual perception of color matching in single-shade material becomes unpredictable when teeth have lower values and higher chroma [2]. Accordingly, it can be suggested that single-shade material tends to show acceptable color matching to teeth with lighter color values, while multi-shade restoration is best selected in esthetic cases of teeth with lower color values and darker shades.

Tooth color is primarily influenced by dentin, while enamel has an effect on optical characteristics such as translucency, opalescence, and color value [20]. Physiological aging, changes in tooth structure as a result of variation in mineral composition, and wear on the enamel can alter light transmission, which affects tooth shade and color-matching of restoration among people and over time [21]. The phenomenon of color matching of restorations to the surrounding substrate is complex, and the subjective visual perception of color difference is influenced by many factors [17]. For instance, resin composition and the size and distribution of fillers affect how light interacts with restorations. Omnichroma relies on the concept of structural color rather than pigments to match the tooth shade. Its uniformly sized and evenly dispersed supra-nano spherical fillers, its relatively high translucency, and the matching RI of resin and fillers result in the amplification of specific wavelengths of light that reflect color from the surrounding substrate and generate tooth-like color [13]. However, this material lacks Bisphenol A diglycidyl dimethacrylate (Bis-GMA) resin (which enhances translucency) and metal oxides (which provide color and enhance opalescence) [2]. Such compositional modifications may alter the optical properties and appearance [2]. Furthermore, the geometry, size, and depth of the cavity and the orientation of enamel prisms at different parts of the tooth affect light scattering and penetration, which becomes limited in deeper cavities, which are more likely to have darker shades of dentin, resulting in a compromised color match [17]. Additionally, cavity size influences the visual perception of resin restorations. Human vision is more able to detect color differences in larger restorations [22].

Color stability is essential for determining the success of dental restorations. Carbonated soft drinks and coffee, frequently consumed in the modern diet, are recognized as potential causes of discoloration [23,24]. The second hypothesis was partially rejected based on observations of the statistically significant effect of staining substances on mean ΔE within and across staining groups, but the color change was comparable between both materials after staining. Some prior studies found that OM restorations showed greater color change after exposure to various staining media than conventional composite [25-27], while others showed that OM material exhibited less color change in comparison to conventional composite [24,28]. The staining resistance and durability of different restorative materials are influenced by water sorption, solubility, and chemical properties, which vary between materials [29]. The literature also showed that the solubility and color stability of OM material are significantly influenced by thermocycling aging [3].

The third hypothesis was also rejected as the RI of single-shade and conventional restorative materials were different. The translucency of a restorative material is a critical factor in determining the amount of light passing through its structure and reflecting the color of the surrounding substrate to produce a tooth-like appearance [30]. A mismatch of RI between components of a restorative material (resin and fillers) as well as between a material and tooth structure results in reducing translucency and affecting light interaction and color matching [30]. Compared with values of refractive indices of enamel and dentin (1.63 and 1.54, respectively) [30], the FT material (body shades used) showed better matching of RI to dentin than OM, contributing to better blending of FT material with tooth color, which is determined mainly by dentin [20].

A combination of visual and instrumental assessments of color-match properties was performed in the present study. The spectrophotometer is an accurate, objective method of measuring color with high precision and sensitivity [3]. However, visual assessment is the most common method of shade selection and assessment in clinical practice; nonetheless, it has the disadvantage of subjectivity. The inter- and intra-evaluator calibrations obtained in this study to minimize variability showed highly acceptable agreement. In addition, evaluators were blind to the type of restoration being evaluated during the visual assessment of color matching to reduce observer bias. To minimize the influence of polychromatic tooth structural variation between teeth collected from different patients of a wide range of ages and shades, a split experimental design was selected in this study. Each extracted tooth received two restorations (OM and FT) randomly on either buccal or lingual surfaces with a standardized cavity design and measure. In addition, the use of extracted teeth in the present study provides better data, considering the nature of the enamel-dentin complex, which can overcome the limitations associated with acrylic teeth like those used in previous similar studies.

However, the results obtained from in vitro studies do not accurately reflect the intraoral environment. Extracted teeth may have structural alterations that negatively influence their optical properties [5]. Color stability was tested to simulate the staining effect of dietary habits (chemical aging), but no mechanical or thermal aging was applied, which would affect the properties of the materials and alter the results [3]. Only three tooth shades were selected for color-match evaluation. Other shades and other optical characteristics of single-shade materials, such as translucency and opalescence, were not investigated in this study and can be explored in the future. Because exploratory in vitro studies provide only preliminary material data, clinical studies are also needed to evaluate the performance of the new material in various clinical situations.

## Conclusions

Based on the results and within the limitations of the current study, it can be concluded that the color-matching ability of single-shade restorations was less than that of conventional composite resin and affected significantly by tooth shade. The color mismatch of single-shade restorations was significantly higher in tooth shade A3.5 than in lighter teeth (B2 and A3). The color stability of this new material was significantly influenced by black coffee, Arabic coffee, and Coca-Cola compared with the control group (distilled water). However, the color changes of single-shade staining groups were similar to those of conventional staining groups. The highest color change was caused by black coffee. The single-shade composite resin had significantly higher RI than conventional restorative material and dentin but showed a closer measurement to the RI of enamel.
